# Electrosprayed Mesenchymal Stromal Cell Extracellular Matrix Nanoparticles Accelerate Cellular Wound Healing and Reduce Gram-Negative Bacterial Growth

**DOI:** 10.3390/pharmaceutics15041277

**Published:** 2023-04-19

**Authors:** Emily N. Wandling, Keera Rhoads, Dennis E. Ohman, Rebecca L. Heise

**Affiliations:** 1 Department of Biomedical Engineering, Virginia Commonwealth University, Richmond, VA 23219, USA; 2 Department of Microbiology and Immunology, Virginia Commonwealth University, Richmond, VA 23298, USA; 3 Research Service, McGuire Veterans Affairs Medical Center, Richmond, VA 23249, USA

**Keywords:** mesenchymal stromal cells, extracellular matrix, nanoparticles, acute respiratory distress syndrome, ventilator-associated pneumonia, antimicrobial

## Abstract

Treatments for acute respiratory distress syndrome are still unavailable, and the prevalence of the disease has only increased due to the COVID-19 pandemic. Mechanical ventilation regimens are still utilized to support declining lung function but also contribute to lung damage and increase the risk for bacterial infection. The anti-inflammatory and pro-regenerative abilities of mesenchymal stromal cells (MSCs) have shown to be a promising therapy for ARDS. We propose to utilize the regenerative effects of MSCs and the extracellular matrix (ECM) in a nanoparticle. Our mouse MSC (MMSC) ECM nanoparticles were characterized using size, zeta potential, and mass spectrometry to evaluate their potential as pro-regenerative and antimicrobial treatments. The nanoparticles had an average size of 273.4 nm (±25.6) and possessed a negative zeta potential, allowing them to surpass defenses and reach the distal regions of the lung. It was found that the MMSC ECM nanoparticles are biocompatible with mouse lung epithelial cells and MMSCs, increasing the wound healing rate of human lung fibroblasts while also inhibiting the growth of *Pseudomonas aeruginosa*, a common lung pathogen. Our MMSC ECM nanoparticles display characteristics of healing injured lungs while preventing bacterial infection, which can increase recovery time.

## 1. Introduction

Acute lung injury (ALI) and the more severe acute respiratory distress syndrome (ARDS) remain life-threatening conditions for patients worldwide. ARDS is categorized into three stages: exudative, proliferative, and fibrotic, depending on disease progression [1]. ARDS is characterized by an influx of inflammatory cells as a response to an injured lung. The inflammatory response leads to progressive alveolar damage and increased fluid, protein, neutrophil, and blood cell permeability in the lung epithelium [2]. Over time, the damage causes necrosis of lung epithelial cells, resulting in gaps in the epithelial barrier, which lowers the efficiency of gas exchange [3]. The progressive injury of the lung due to the inflammatory response leads to pulmonary edema, hypoxemia, and pulmonary fibrosis and can ultimately culminate in respiratory failure [4]. 

ARDS is most prevalent in critically ill patients with comorbidities, including sepsis and pneumonia. There are approximately three million patients with ARDS every year. Out of these causes, 10% consist of patients in the intensive care unit [5]. The mortality rate of ARDS continues to remain high at 43% [6]. The frequency of ARDS in critically ill patients has only continued to increase due to the coronavirus, SARS-CoV-2, pandemic that began in 2019 [7].

Currently, there are not any pharmacological treatments to cure ARDS [8]. Treatments and care regimens are used to mitigate symptoms, such as mechanical ventilation to supply oxygen to the patient’s airspaces, anti-inflammatory medications, antibiotics to treat infection, diuretics to remove excess fluid from the lungs, and sedative medications to relieve pain from shortness of breath [9]. Approximately 24% of ARDS patients receive mechanical ventilation treatment, which can contribute to an already inflammatory lung environment [10]. Mechanical ventilation may also induce ventilator-associated pneumonia (VAP) from colonization of an inhaled microbial pathogen in the lower respiratory tract [11]. 

Due to the increased use of antibiotics in medical care, there has been a case of antibiotic resistance for almost every antibiotic that has been used as a treatment. Acquiring an infection with an antibiotic-resistant bacterium is known to increase the mortality rate, the need for intensive care treatment, and healthcare costs for the patient [12]. Antimicrobial resistance has reportedly risen during the COVID-19 pandemic for *Pseudomonas aeruginosa*, *Staphylococcus aureus*, and *Klebsiella pneumoniae*, common pathogens involved in VAP [13]. Out of the VAP diagnoses from COVID-19, nearly 66.67% of infections were caused by multi-drug resistant pathogens [14]. This alarming increase in antibiotic-resistant bacterial strains shows there is an urgent need to develop novel therapies to prevent lung infection and damage.

To combat lung injury from ARDS, mechanical ventilation, and VAP, our proposed solution is to develop nanoparticles from the extracellular matrix (ECM) of mesenchymal stromal cells (MSCs) that can stimulate tissue healing and prevent bacterial infection. Previously, ECM from pig lungs (PL) has been harvested and developed into a nanoparticle treatment [15]. The PL ECM nanoparticles were also able to modulate a pro-regenerative phenotype macrophage after introduction to murine bone marrow-derived monocytes. Nanoparticles were chosen as the method of delivery for ECM for both their size and charge characteristics to reach the distal regions of the lungs while avoiding mucosal fluid in the inflamed lung [15].

In this study, marrow-derived mesenchymal stromal cells (MMSCs) were our choice of source material for ECM due to their secretome, healing abilities, and ability to polarize macrophages. The MSC secretome, all of the molecules released by a cell, includes cytokines, mRNAs, extracellular vesicles, growth factors, and lipid molecules [16]. The utilization of the MSC secretome for treatment is beneficial as it is a cell-free biomaterial, lowering the risk of rejection by the immune system [17]. These molecules can impact cell-cell signaling by inducing a physiological response, including stimulating the production of ECM, suppressing apoptosis, and activating anti-fibrotic, anti-inflammatory, or angiogenic responses [18]. In vitro studies have shown that the secretome can affect several different types of both innate and adaptive immune cells, including macrophages, neutrophils, and T and B lymphocytes [19]. Additionally, MSCs have displayed potential to mediate the effects of ARDS by preserving the alveolar epithelial barrier in models of ARDS and increasing the clearance of alveolar fluid in the inflamed lung. In fact, the administration of MSCs as a therapy in human ARDs patients improves patient survival rates due to the anti-inflammatory, tissue repair, and antimicrobial properties of the MSCs [20]. 

Another important molecule present in the MSC secretome is antimicrobial peptides (AMPs). AMPs inhibit the growth of bacteria through depolarization of the bacterial cell membrane and slow the formation of the protective biofilm layer for *P. aeruginosa* and *S. aureus* [21]. Some of these bioactive peptides have been identified, including tumstastin, derived from collagen IV, which can increase fibroblast migration and proliferation, and the peptide DGGRYY, from collagen I, which activates neutrophils. Other peptides have not been identified by name, such as peptide fragments from elastin, which are able to encourage fibroblast migration [22]. Peptides formed from degraded fibronectin, laminin, and vitronectin have been proven to have antimicrobial activity against both Gram-positive *S. aureus* and Gram-negative *Escherichia coli* and *P. aeruginosa* [23]. All of these factors make the MSC secretome a viable candidate as a biomaterial for nanoparticles. 

Our goal was to harness the pro-regenerative and antimicrobial properties of the MMSC ECM into the size of a nanoparticle that could reach the distal region of the lung. Decellularized ECM (dECM) proteins, including collagen, glycosaminoglycans, and growth factors, increase cellular proliferation without eliciting an immune response. The MSC secretome releases cytokines and peptides that activate the proliferation of progenitor stem cells, stimulate the formation of new ECM, suppress apoptosis and fibrosis, and inhibit bacterial growth [24,25]. The nanoparticle size will allow the treatment to reach the distal region of the lungs. The negative zeta potential of the nanoparticles will avoid the deposition of mucus in the lung epithelial barrier [15]. We hypothesize that the size of the nanoparticles and the pro-regenerative effects of the MMSC ECM will combine to form a nanoparticle treatment that can reach the distal region of the lungs and stimulate lung tissue growth after injury. Additionally, we believe there will be antibacterial peptides found in our MMSC ECM nanoparticles that can inhibit the growth of both gram-positive and gram-negative bacteria. 

## 2. Materials and Methods

### 2.1. Mammalian Cell Culture and Media Preparation 

A cell line of mouse bone marrow-derived mesenchymal stromal cells (MMSCs, Gibco, Thermofisher, Grand Island, NY USA) was grown on cell culture flasks (T75 and T175, Greiner, Thermofisher, Grand Island, NY USA) to obtain decellularized ECM (dECM). Cell seeding density was 500,000 cells for the T-75 flask and 2,000,000 cells for the T-175 flask. MMSCs were cultured in Dulbecco’s Modified Eagle Medium (DMEM, Gibco) modified with GlutaMAX supplement (Gibco). The media were supplemented with 10% fetal bovine serum qualified for mesenchymal stem cells (FBS, Hyclone, Thermofisher, Grand Island, NY USA) and 250 µL of Gentamicin (Gibco). Cell culture conditions were set at an atmosphere of 5% CO_2_ at 37 °C. The culture medium was changed every 2–3 days. 

Mouse Lung Epithelial (MLE-12, ATCC, Manassas, VA USA) cells were grown in Dulbecco’s Modified Eagle (Gibco) and supplemented with 2.5 mL of 100 mM sodium pyruvate (Sigma Life Sciences, Thermofisher, Grand Island, NY, USA), 0.6 g of sodium bicarbonate (Flinn Scientific, Thermofisher, Grand Island, NY, USA), 250 µL of 10 mg/mL insulin (Gemini Bio-Products, West Sacramento, CA USA), 5 mg of transferrin (Sigma-Aldrich, St. Louis, MO, USA), 5.2 µL of 0.5 mg/mL sodium selenite (Lonza, Basel, Switzerland), 50 µL of 100 µM hydrocortisone (Lonza), 50 µL of 100 µM β-estradiol (Lonza), 5 mL of 200 mM L-glutamine (Quality Biological, Thermofisher, Grand Island, NY, USA), and 10 mL of FBS (Gibco).

Human fibroblast cells (NHLF, Lonza) were cultured in fibroblast basal medium (FBM, Lonza) with a growth supplement package (FGM2 SingleQuots, Lonza). 

### 2.2. Decellularization of MMSCs with 0.1% Sodium Deoxycholate and DNase I 

MMSCs were seeded on tissue culture flasks at a density of 500,000 cells for a T75 flask and 2 million cells for a T175 flask. Cells were cultured until 90–95% confluency, which was typically three-five days after initial cell seeding. The MMSC cell layer was washed five times with 1X phosphate-buffered saline (PBS). A solution of 0.1% sodium deoxycholate was added to the cell flask and incubated for three minutes at 37 °C. The ECM sheet was then collected and washed with Hank’s Balanced Salt Solution (HBSS, Gibco) three times. Tissue was centrifuged at 300 rcf for 2 min to remove the wash solution as needed if tissue was difficult to collect. The ECM tissue was submerged in a solution of DNase I (50 U/mL, Sigma-Aldrich) and incubated at 37 °C for 30 min. The DNase I solution was then removed, and the dECM was washed with PBS three more times, centrifuging at 300 rcf for two minutes to collect the supernatant if needed. dECM can be stored for up to 4 months at 4 °C in 2 mL of PBS and 2 mL of Antibiotic-Antimycotic Solution (10,000 U/mL Penicillin, 10,000 µg/mL Streptomycin, 25 µg/mL Fungizone, Thermofisher, Grand Island, NY, USA) [26]. Before nanoparticle fabrication, the decellularized ECM tissue was lyophilized into a powder. The powder was formed after a lyophilization cycle of 36 h and then stored at −20 °C for long-term storage. 

### 2.3. MMSC ECM Nanoparticles Fabrication Using Electrospray Deposition 

**Figure 1:**
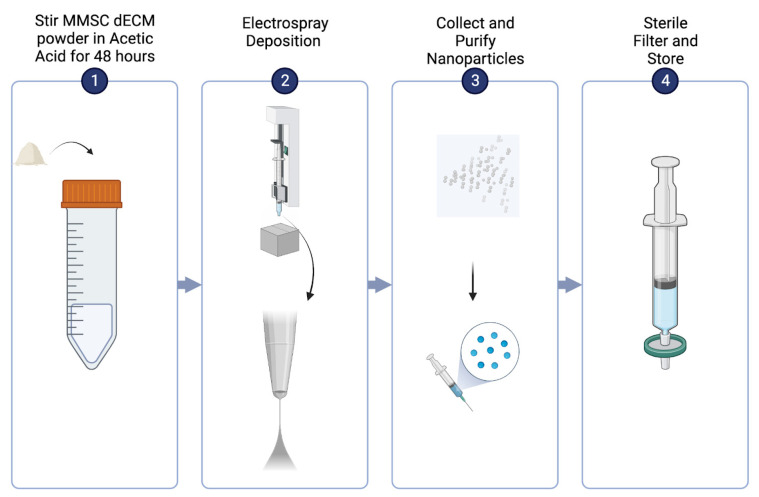
Electrospray deposition procedure.

Decellularized MMSC ECM powder (50 mg) was dissolved in 80% *v*/*v* (*volume*/*volume*) of glacial acetic acid and stirred for 48 h at room temperature (Figure 1). After stirring, the ECM-acetic acid solution was drawn up by a syringe with a 26-gauge blunt-tip needle. The needle was placed in a syringe pump and set to a flow rate of 0.6 mL/hour. Cables from the voltmeter were attached to the top of the 26-gauge needle and a piece of aluminum foil to complete the electrospray system. The working distance between the stationary aluminum foil and needle was 8 cm. The voltmeter was set to −15 kV, and a syringe pump was started and allowed to run overnight.

Once the solution was deposited on the foil, the foil was sprayed with 70% ethanol, and the ECM nanoparticle residue was collected into a solution. The nanoparticles were sorted into the correct size by drawing the solution through a series of needles: 18-gauge, 26-gauge, and then 27-gauge. The nanoparticle solution was then purified using a 0.45-um pore-size filter. The nanoparticle solution was added to 10 mL of distilled water. The nanoparticle solution was stored at −80 °C or lyophilized for 36 h into a powder. 

### 2.4. Mass Spectrometry

Mass spectrometry was completed with the assistance of the Biomolecular Analysis Facility at the University of Virginia. The lyophilized MMSC ECM nanoparticle samples were suspended in 1 mL of water (LC-MS grade), 9 mL of cold methanol, and 1 mL of cold acetone. The samples were allowed to precipitate overnight at −80 °C. The next day, samples were centrifuged at 4 °C for 2 h at 3000 rpm. The pellets that were obtained were washed with 1 mL of cold methanol and followed by 20 min of centrifugation at 15,000 rpm for a total of 3 times. Protein pellets were dried and then suspended in 50 µL of 0.1 M ammonium bicarbonate and reduced using 10 mM dithiothreitol at room temperature for 1 h. Then, 50 mM iodoacetamide was added and incubated in the dark at room temperature for 30 min. The sample was then digested overnight at 37 °C with 0.5 µg of Trypsin. For sample purification, C18 column tips were utilized. The samples were then dried in a speed vacuum and added to 0.1% of formic acid. An amount of 5 µL of each sample was injected to obtain the mass spectrum. 

Scaffold 5 was used for mass spectrometry analysis. Peptide identifications were accepted if they could be established at a greater than 95.0% probability and then proteins were sorted based on function, locality, and type of ECM. PeptideRanker was then used to determine if ECM proteins and peptides were potentially bioactive. Scores were on a scale of 0–1, with 1 being the highest probability of having bioactive properties [27].

### 2.5. Nanoparticle Size and Charge Characterization

The size of the MMSC ECM nanoparticles was determined using Scanning Electron Microscopy [SEM]. A sample of aluminum foil containing electrospray nanoparticles was affixed to a silicon wafer and coated with platinum to obtain the images. A Malvern Zetasizer [ZN90] was utilized to obtain both the size and zeta potential of the MMSC ECM nanoparticles. 

### 2.6. MTT Assay 

An MTT assay was conducted to evaluate the biocompatibiliy of the MMSC ECM nanoparticles. In vitro cytotoxicity testing was critical to determine if the nanoparticles were toxic to cells before using them as a treatment in an in vivo model. The cytotoxic activity of the nanoparticles were evaluated on MMSCs and MLE-12 cells. MMSCs were tested to ensure that the nanoparticles would not affect the population of stem cells in the lungs, as stem cells help contribute to tissue repair and can differentiate into epithelial cells [28]. MLE-12 cells were used in the assay to ensure that the nanoparticles would not induce necrosis in the lung epithelial layer. 

To evaluate the cytotoxic effects of the nanoparticles, cells were seeded at a density of 10,000 per well and grown in a 96-well plate (Falcon, Thermofisher, Grand Island, NY USA) for 48 h. Media were then replaced with nanoparticle treatments with concentrations of 0.5 ng/mL, 1.0 ng/mL, 2.0 ng/mL, and 4.0 ng/mL, or replenished with new media as the control. Treatments were added for 24 h and then the procedure from the MTT assay was conducted (Roche, Basel, Switzerland). 

To determine if ECM nanoparticles were biocompatible, ECM nanoparticle treatment, in concentrations of 0.5 ng/mL, 1.0 ng/mL, 2.0 ng/mL, and 4.0 ng/mL, were added to 10,000 cells in a 96-well plate (Falcon) at the same time. This was repeated with cell culture media, conditioned media with secreted ECM proteins, and 1.92% ethanol solution as controls. The cells were grown for 24 h and then an MTT assay (Roche) procedure was followed. 

### 2.7. Scratch Assay 

Human lung fibroblast cells were utilized for the scratch assay to evaluate if the ECM nanoparticles stimulate wound healing. Fibroblasts are critical in the healing process, as they secrete growth factors, cytokines, and ECM to repair an injury [29]. A total of 200,000 cells were seeded into a 6-well cell culture plate (Falcon), which was marked with a horizontal line on the bottom using a sterile straight edge. Cells were incubated at 37 °C to grow overnight. 

The next day a 20 µL pipette tip was used to scratch the cell layer perpendicular to the horizontal such as at the bottom of the well. The media were aspirated from each well and then washed with 1 mL HEPES solution (Gibco). Media supplemented with nanoparticles in concentrations of 0.50 ng/mL, 1.0 ng/mL, 2.0 ng/mL, and 4.0 ng/mL, were then applied. Media without any nanoparticles was used as a control. Once the media were applied, the wells were imaged using a phase contrast microscope to determine the initial area of the scratch. Pictures were taken above and below the horizontal line applied on the well. The well plate was incubated for 48 h after the scratch to determine the closure rate, pictures were taken every 24 h. Once images were collected, ImageJ software was used to determine the area of the scratch [30]. The wound healing rate for each scratched cell was calculated using the following equation:

Percentage of Wound Healed = [Area(T_0_) − Area(T_X_)]/Area(T_0_).

Area(T_0_) was the initial denuded area for each image location. Area(T_X_) was the denuded area for each location after 24 or 48 h.

### 2.8. Bacterial Assays 

An overnight culture was prepared by taking a colony of *Escherichia coli* HfrH from a LB agar plate and inoculating it in 5 mL of LB broth. The culture was incubated overnight in a shaking incubator at 37 °C and 200 rpm. The following day, 100 µL of that overnight culture was added to 10 mL of LB broth. MMSC ECM nanoparticles were added in concentrations of 4.0 ng/mL and 0.50 ng/mL to the culture. An LB broth control without nanoparticles was used as a control. Flasks were shaken overnight at 200 rpm at 37 °C. At the time points of 1 h, 3 h, 6 h, and 24 h, 100 µL of the culture was taken, serially diluted, and spread onto an agar plate, which was incubated overnight at 37 °C. The next day, colonies grown on the plate were counted and analyzed for colony forming units (CFU). The procedure was utilized for testing if the nanoparticles inhibit the growth of *Staphylococcus aureus* Newman and *Pseudomonas aeruginosa* PA01.

### 2.9. Statistical Analysis 

All statistical analyses were performed using Prism 9 (GraphPad). Ordinary analyses of variance with multiple comparisons were used to determine the significance of the results. Significant results were determined to have a *p*-value < 0.05. 

## 3. Results

### 3.1. Mass Spectrometry

**Figure 2:**
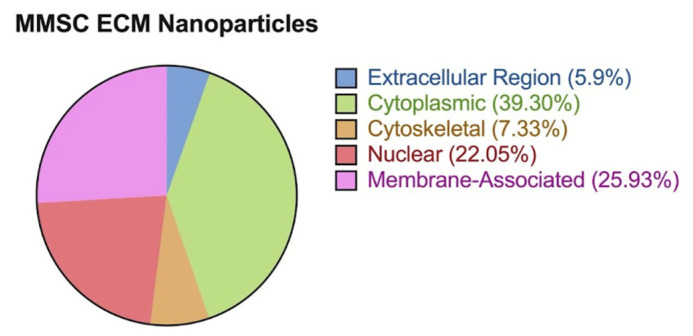
Comparison of the location of ECM proteins from the MMSC ECM nanoparticles.

**Figure 3:**
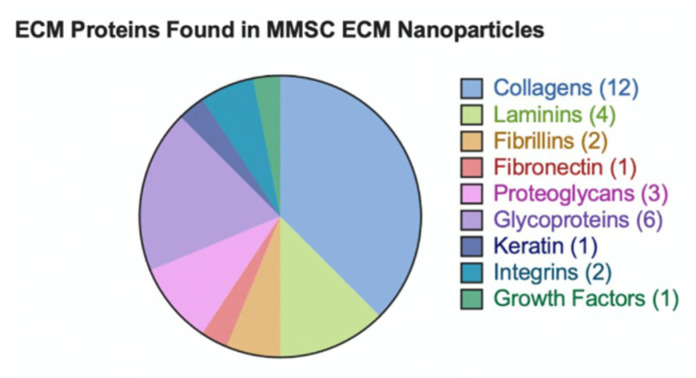
Categorization of ECM proteins found in the MMSC ECM nanoparticles.

The mass spectrometry results confirmed that both the MMSC ECM nanoparticles contained important structural ECM proteins and potential proteins and peptides that contribute to their bioactivity. The nanoparticles had the greatest percentage of proteins from the cytoplasm and then membrane-associated proteins (Figure 2). Scaffold 5 determined that 160 proteins from the total proteasome were from the ECM region in the MMSC ECM nanoparticles. Collagens, laminins, fibronectin, proteoglycans, glycoproteins, growth factors, integrins, keratin, and fibrillins were all predicted to be in the MMSC ECM nanoparticles as seen in (Figure 3). 

PeptideRanker indicated that the MMSC ECM nanoparticles were composed of several protein subunits that potentially could possess bioactivity, including peptide sequences derived from collagen, fibronectin, and laminin (Table 1). In particular, cationic sequences from fibronectin and the beta-1 chain of laminin are known to have antimicrobial effects. The membrane protein tetraspanin was found in the ECM proteins, which have been shown to have antimicrobial activity. Thrombospondin-1 and thrombospondin-2 were found as well. Hydrophobic regions from thrombospondins have been proven to have antimicrobial activity against both gram-positive and gram-negative bacteria [23]. Glycoprotein fibulin-1 was found in the MMSC ECM nanoparticles known to encourage lung fibroblast proliferation and attachment [31]. 

### 3.2. Nanoparticle Size and Charge Characteristics

**Figure 4:**
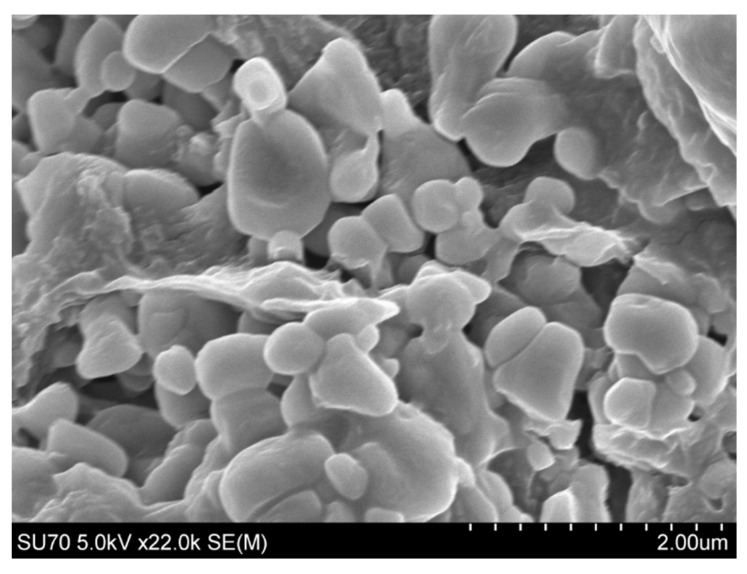
SEM image of deposited MMSC ECM electrosprayed nanoparticles on aluminum foil before purification.

**Figure 5:**
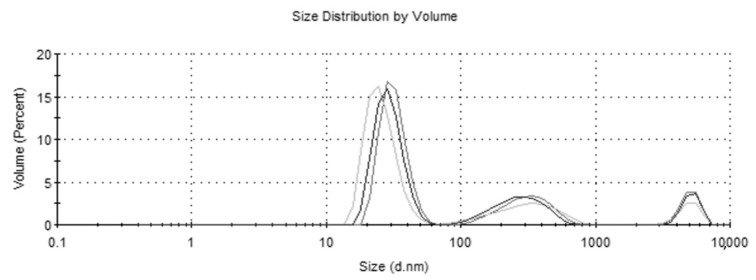
Size distribution graph of MMSC ECM nanoparticles. Sample was run three times and plotted in the figure above.

The MMSC ECM nanoparticles had an average size of 273.4 nm (±25.6) and a zeta potential of −11.17 (±0.611 (Figure 4 and Figure 5). The MMSC ECM nanoparticles were smaller than 300 nm, meaning that they are capable of reaching the distal alveolar regions of the lungs [15]. They had a negative zeta potential, which may help prevent the aggregation of nanoparticles and attachment to mucus once delivered to the lung [32,33]. The negative charge may help the nanoparticles evade detection by phagocytic cells and improve biocompatibility tested by the MTT assay [34].

### 3.3. MTT Assay

**Figure 6:**
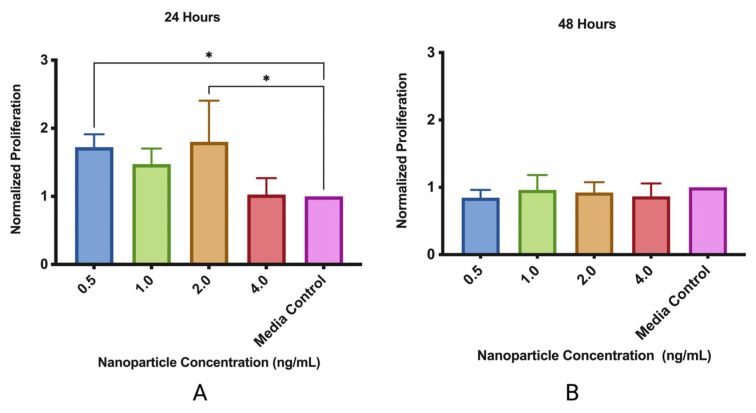
MMSC ECM nanoparticles are biocompatible. (**A**) MMSC ECM nanoparticles were added with MLE-12 cells for 24 h to test proliferative effects; (**B**) MMSC ECM nanoparticles were added to MLE-12 cells for 48 h to test cytotoxicity. Data displayed is the mean ± standard deviation. * indicates *p* < 0.05. N = 3.

**Figure 7:**
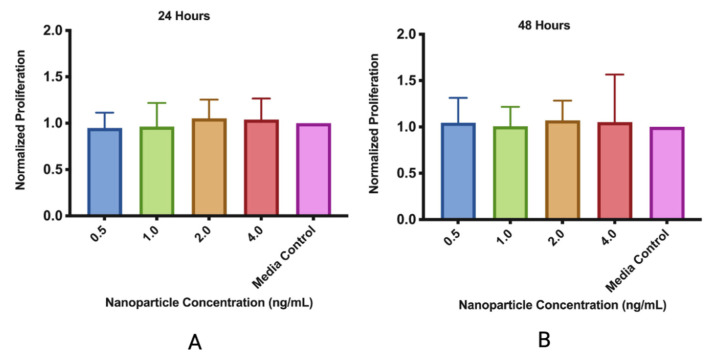
MMSC ECM nanoparticles are biocompatible. (**A**) MMSC ECM nanoparticles were added with MMSCs for 24 h to test proliferative effects. (**B**) MMSC ECM nanoparticles were added to MMSCs for 48 h to test cytotoxicity. The data displayed are the mean ± standard deviation. N = 3.

The MMSC ECM nanoparticles were determined to be biocompatible with the mouse lung epithelial cells (Figure 6). Both nanoparticle concentrations of 0.5 ng/mL and 1.0 ng/mL significantly increased cellular viability, as they were both approximately 80% more confluent than the media control well. After 48 h, we saw that the nanoparticles did not significantly affect cell viability. For the MMSCs, the MMSC ECM nanoparticles were concluded to be non-cytotoxic (Figure 7). 

### 3.4. Scratch Assay

**Figure 8:**
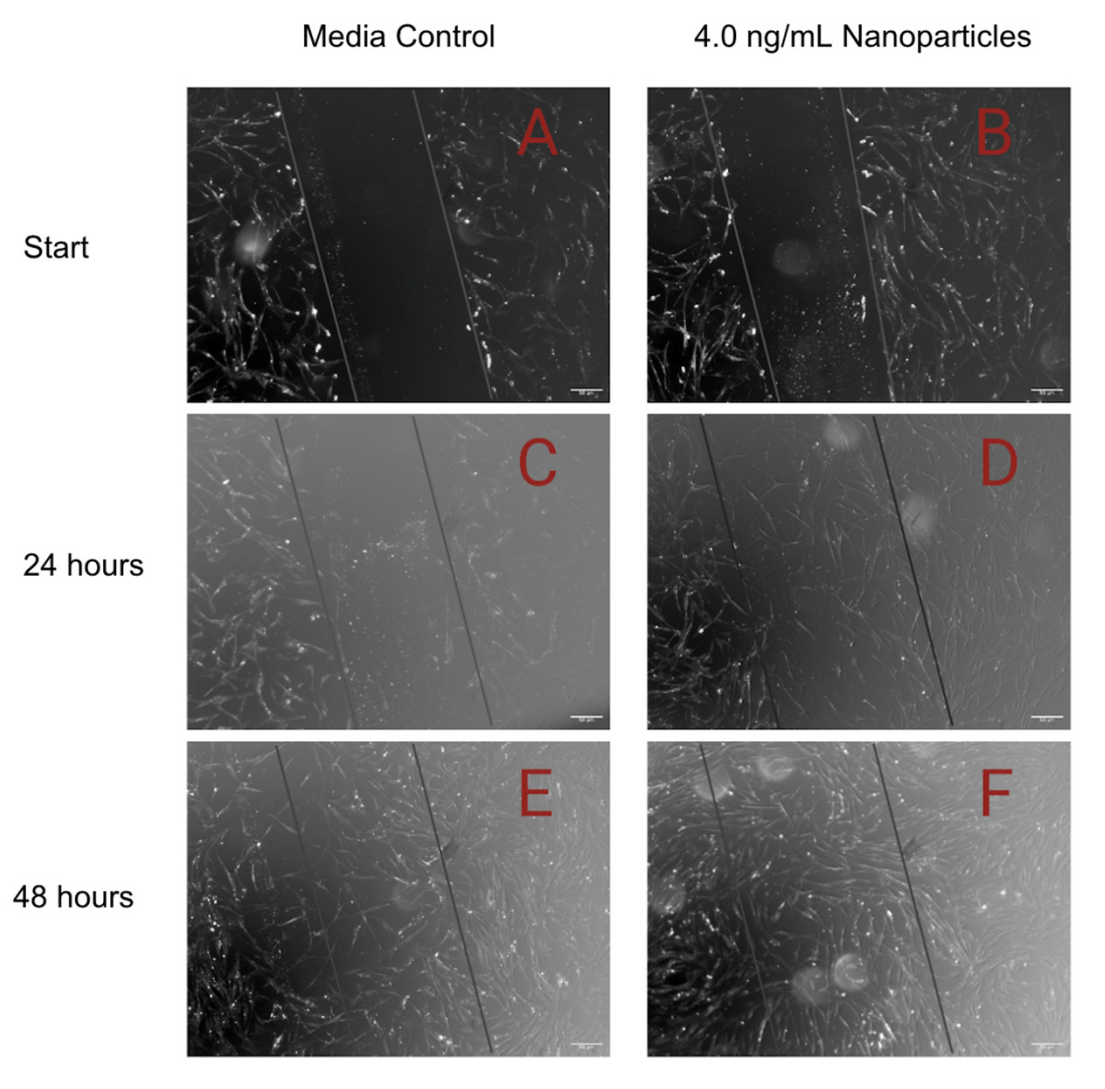
Scratch assay images of human lung fibroblasts. Confluent cell sheets were scratched and replenished with media [**left**] or 4.0 ng/mL of MMSC ECM nanoparticles [**right**]. The outline of the original scratch is superimposed on each image. Images obtained every 24 h. The scratch was closed at a faster rate with supplementation of nanoparticles. (**A**) Media, 0 h; (**B**) 4.0 ng/mL nanoparticles, 0 h; (**C**) media, 24 h; (**D**) 4.0 ng/mL nanoparticles, 24 h; (**E**) media, 48 h; (**F**) 4.0 ng/mL nanoparticles, 48 h. (magnification: 50 µm).

**Figure 9:**
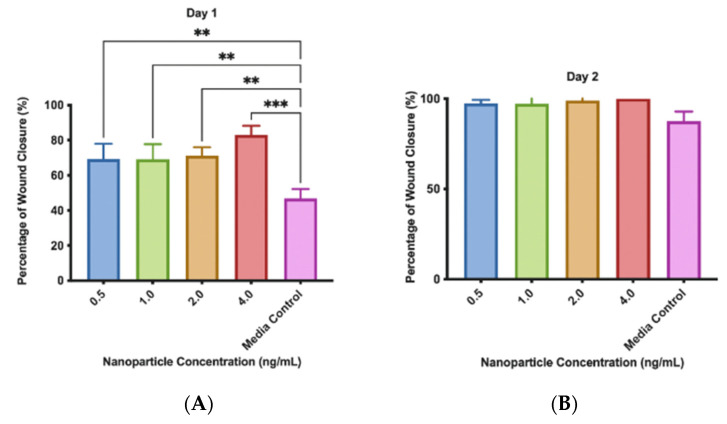
Scratch assay results. Fibroblasts supplemented with MMSC ECM nanoparticles have a faster wound closure rate compared to the media control. (**A**) Fibroblast growth rate after 24 h with nanoparticle media. (**B**) Fibroblast growth rate after 48 h with nanoparticle media. The data displayed are the mean ± standard deviation. ** indicates *p* < 0.001, *** indicates *p* < 0.0001, N = 3.

The rate of wound closure was significantly increased when MMSC ECM nanoparticles were added to the scratched fibroblast cell layer (Figure 8 and Figure 9). After 24 h, media with nanoparticles in a concentration of 4 ng/mL had the greatest effect on the healing of the scratch, as it increased the closure rate of the scratch by 80% compared to the media control. We found that as the concentration of nanoparticles increased in the media, the rate of scratch closure increased as well. After 48 h, the scratches from the nanoparticle treatment groups were closed while the scratch supplemented with the media control was, on average, 87% healed.

### 3.5. Bacterial Assays

**Figure 10:**
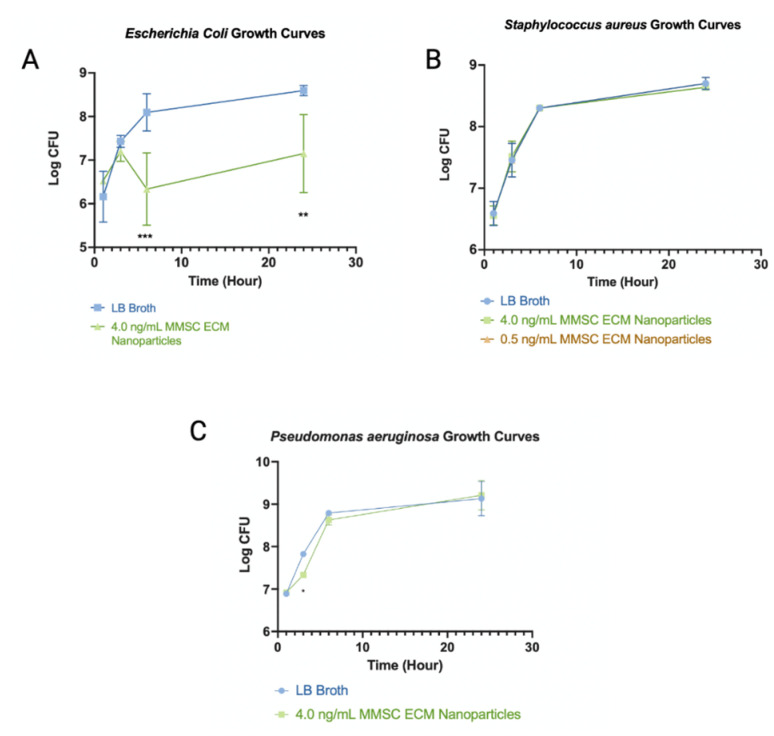
Bacterial assay growth curves. (**A**) Comparison of *E. coli* growth curves for LB Broth control culture and 4.0 ng/mL MMSC ECM nanoparticle culture. MMSC ECM nanoparticles show significant inhibition of growth at hours 6 and 24. (**B**) Comparison of *S. aureus* growth curves for LB broth control culture, 4.0 ng/mL MMSC ECM nanoparticle culture, and 0.5 ng/mL MMSC ECM nanoparticle culture. MMSC ECM nanoparticles did not inhibit the growth of *S. aureus*. (**C**) Comparison of *P. aeruginosa* growth curves for LB broth control culture and 4.0 ng/mL MMSC ECM nanoparticle culture. MMSC ECM nanoparticles show significant inhibition of *P. aeruginosa* growth at hour 3. The data displayed are the mean ± standard deviation. * indicates *p* < 0.05, ** indicates *p* < 0.001, *** indicates *p* < 0.0001. The concentration of 4.0 ng/mL MMSC ECM nanoparticles was found to be significantly different after 3 h of incubation. N = 3.

MMSC ECM nanoparticles were able to inhibit the growth of gram-negative *E. coli* and *P. aeruginosa* wild-type strains (Figure 10). MMSC ECM nanoparticles inhibited growth at a concentration of 4.0 ng/mL. Nanoparticles significantly reduced bacterial growth of *E. coli* after 6 and 24 h of culture incubation. It was found that *S. aureus* growth was not significantly reduced when the MMSC ECM nanoparticles were added to the culture. MMSC ECM nanoparticles significantly reduced *P. aeruginosa* growth at 3 h.

## 4. Discussion

### 4.1. MMSC ECM Nanoparticle Protein Characterization 

It was determined that the MMSC ECM nanoparticles are capable of inducing a pro-regenerative cellular response while also inhibiting the growth of bacteria. Mass spectrometry results confirmed that characteristic ECM proteins were retained during the decellularization process with sodium deoxycholate and 80% acetic acid digestion for electrospray deposition. This was shown by the fact that collagens, laminins, fibronectin, glycoproteins, and growth factors were present in both samples. 

The MMSC ECM also had a more diverse proteasome including integrins, fibrillins, and keratins. The microfibrils in the ECM are composed of fibrillin, proving structural integrity and scaffolding for organ systems. Fibrillin is also critical in participating in the development of elastin fibrils [35]. Keratin has been shown to improve cell attachment and proliferation of fibroblasts and adipose-derived stem cells [36]. Both fibrillin and keratin may help stimulate a wound-healing response in the injured lung by inducing the formation of new ECM by increasing cell attachment and deposition of ECM scaffolding proteins. Another glycoprotein thrombospondin can interact with ECM structural components, growth factors, cytokines, and matricellular proteins. This includes interactions with matrix-metalloproteinase [MMP] 2 and MMP9 that can regulate collagen homeostasis and prevent the overproduction of collagen that can form fibrotic tissue [37]. 

Additionally, the glycoproteins fibulin-1 and fibilin-2 were found in the MMSC ECM nanoparticles. Fibilins are hypothesized to organize ECM structural fibers, including the basement membrane and elastin fibers. Fibulin 1 has been found to organize ECM fibers composed of elastin and fibrillin 1 and 2 during murine lung development. Fibulin-2 can bind to elastin and fibrillin 1, serving to attach microfibrils to elastin [38]. Fibulin-5 mediates the crosslinking of tropoelastin monomers into insoluble elastin polymers and is typically found in higher levels in the lungs [35]. The presence of fibilin found in the ECM nanoparticles may help repair injured ECM in the wounded lung. 

### 4.2. Biocompatibility and Wound Healing Effects

Furthermore, the MTT assay results indicated that the MMSC ECM nanoparticles are not cytotoxic and promote wound healing. For MLE-12 cells, nanoparticles did not affect cellular viability after 24 h of exposure to the MMSC ECM nanoparticles. Once nanoparticles were applied to a pre-seeded epithelial cell layer that was grown for 48 h, cell count was slightly increased compared to media alone, which indicated that the nanoparticles did not induce cell death. These promising results, suggest that when the nanoparticle interacts with the lung epithelium, the ECM nanoparticles will not induce cell death further injuring wounded tissue. 

A similar effect occurred when the MMSC ECM nanoparticles were added to MMSCs. However, higher concentrations of nanoparticles increased the cell count of MMSCs while nanoparticles added at a lower concentration increased the cell count of MLE-12 cells. This may be due to the ability of MSC secretome, in the form of dECM, to encourage the growth of MSCs as we are adding their own cellular product into the media to support growth [24]. Our ECM nanoparticles may be about to support MSCs in the lung. When applied to an injured lung, the nanoparticles may help activate the lung MSCs which can then work to issue a wound-healing response and regenerate lung tissue. 

We showed that our nanoparticles encourage wound closure when applied to lung fibroblasts. All concentrations of our MMSC ECM nanoparticles closed the wound at a significantly faster rate than the media alone. An increased tissue repair rate may be critical for a patient’s condition, as longer healing times may further aggravate the inflamed lung environment. Fibroblasts are the main cellular source of the ECM, so the repair of lung fibroblasts is critical to supporting new cells and tissue development [39]. Fibroblasts serve another important role in the lung, modulating differentiation, and proliferation of alveolar epithelial cells [40]. Their vitality is then critical to repairing damaged lung epithelium. MSCs have been reported to recruit fibroblasts to an injury site and our MMSC ECM nanoparticles show a similar effect by increasing the rate of wound closure [40]. The ECM protein fragments found in the nanoparticles may help stimulate the production of a new layer of ECM, which may explain the rapid wound closure. 

The MMSC ECM nanoparticles are able to promote cell viability of epithelial cells, mesenchymal stromal cells, and fibroblast cells, all important components of the lung environment. With these promising results in vitro, it may be possible to see the effects of the ECM nanoparticles in an in vivo novel.

### 4.3. MMSC ECM Nanoparticle Anti-Microbial Effects

It was shown here that the ECM nanoparticles have antimicrobial properties. The MMSC ECM nanoparticles were effective at inhibiting the growth of *E. coli* and *P. aeruginosa*. *E. coli* was inhibited after 6 and 24 h of incubation with nanoparticles and *P. aeruginosa* growth was inhibited around 3 h of incubation. The timeframe of growth inhibition of bacteria corresponds to the amount of time it is anticipated that the nanoparticles would remain in the lungs before clearance, approximately 1–6 h. The mass spectrometry analysis revealed some antimicrobial peptides that may be responsible for these effects. 

According to PeptideRanker, the ECM nanoparticles contain peptides that are considered to be bioactive, including collagen fragments, fibronectin, and laminin subunits that ranked highly on the bioactivity scale. These ECM proteins are known to release bioactive peptides upon partial proteolysis. In particular, it is known that sequences with high hydrophobicity or cationic sequences have an antimicrobial effect through interactions with the bacterial cell membrane [23]. 

Thrombospondins, found in the MMSC ECM nanoparticles are another ECM protein that contains a higher concentration of hydrophobic amino acids that can cause an antimicrobial effect in both Gram-positive and Gram-negative bacteria. The nanoparticles also contained the beta-1 chain of laminin, which is composed of highly cationic peptide sequences that can interact with the negative surface charge of the bacteria and induce an antimicrobial effect [23]. Tetraspanin proteins contain two extracellular loops and four hydrophobic transmembrane regions, which may destabilize the bacterial cell membrane as well [41,42]. The presence of these bioactive peptides makes the ECM nanoparticles promising antimicrobial agents on top of being able to stimulate wound healing in the lungs. 

Although it is not fully understood, there are a few mechanisms that propose how antimicrobial peptides inhibit the growth of bacteria. As peptides contain hydrophobic and cationic regions, they can interact with the lipopolysaccharides of the gram-negative membrane or teichoic acid and peptidoglycans in the gram-positive membrane [23]. These interactions can induce conformational changes in the cell wall, causing bacteria cell necrosis [43]. Positive peptide sequences can disrupt the lipid sequence of bacterial membranes by clustering lipids in one region due to the attraction to the cationic peptides, which stops cell growth [44]. The binding of the cationic peptide to a negatively charged lipid can also destabilize the charge of the bacterial cell membrane [43]. Through electrostatic interactions, the peptide can insert itself into the lipid bilayer and form a pore in the structure of the membrane [45]. Instead of permeabilizing the membrane, AMPs can also bind to intracellular targets to stop metabolic processes. AMPs, such as Buforin II, are found to directly bind to DNA and RNA molecules after entering the bacterial cell [46].

## 5. Conclusions

We have developed nanoparticles made from the ECM of MMSCs. MMSC ECM nanoparticles are composed of peptides that induce a regenerative effect to heal injured lung tissue while inhibiting the growth of bacteria. This combination of properties makes them an ideal treatment for ARDS as the nanoparticles are capable of reaching the distal regions of the lungs while preventing bacterial infection if the patient needs mechanical ventilation treatment. For future research, antibiotics can be added to the nanoparticles to increase the efficacy of the treatment against bacteria. Additionally, nanoparticles should be converted into an aerosolized treatment for direct and rapid delivery to injured lung tissue.

## Figures and Tables

**Table 1:** Selected MMSC ECM nanoparticle proteins and their predicted bioactivity ranking from PeptideRanker. Peptides are listed from the highest bioactivity score to the lowest.

ECM Protein	PeptideRanker Bioactivity Score
Laminin subunit alpha-5	0.999916
Laminin subunit beta-1	0.999903
Laminin subunit gamma-1	0.999868
Thrombospondin-1	0.999425
Tetraspanin	0.997189
Thrombospondin-2	0.997116
Fibronectin	0.991198
Collagen alpha-1[XII] chain	0.991148
Collagen alpha-2[IV] chain	0.89322
Collagen alpha-2[I] chain	0.849786
Collagen alpha-1[I] chain	0.849393

## Data Availability

Data will be made available upon request from the corresponding authors.
